# Transforming a U.S. scholarly concentrations program internationally: lessons learned

**DOI:** 10.1186/s12909-019-1545-7

**Published:** 2019-04-25

**Authors:** Stephen M. Sozio, Rümeyza Kazancıoğlu, Fatih Küçükdurmaz, Meliha Meriç Koç, Dilek Sema Arici, Rebecca M. DiBiase, Jeremy A. Greene, Mary Catherine Beach

**Affiliations:** 1 Department of Medicine, Division of Nephrology, Johns Hopkins University School of Medicine, 301 Mason Lord Dr, Suite 2500, Baltimore, MD 21224 USA; 2 Welch Center for Prevention, Epidemiology, and Clinical Research, Johns Hopkins Medical Institutions, Baltimore, MD USA; 3 Bezmiâlem Vakıf University School of Medicine, Istanbul, Turkey; 4 Department of Orthopedics and Traumatology, Marmara University, Istanbul, Turkey; 5 Department of History of Medicine, Johns Hopkins University School of Medicine, Baltimore, MD USA; 6 Berman Institute of Bioethics, Johns Hopkins University School of Medicine, Baltimore, MD USA

**Keywords:** Scholarly concentrations program, Medical student, International education

## Abstract

**Background:**

Scholarly Concentrations programs in U.S. medical schools aim to instill passion for critical thinking and promote careers in academic medicine. The rise of these programs has seen variable goals, structure, and outcomes. Transformation of these programs internationally is in its infancy.

**Methods:**

We describe implementation of the Johns Hopkins School of Medicine Scholarly Concentrations program, offering Basic Science, Clinical Science, Medical Ethics/Healing Arts, History of Medicine, and Public Health/Community Service, at Bezmiâlem Vakif University in Istanbul, Turkey. Over six modules in the preclinical years, students develop a faculty-mentored experience which encourages the acquisition of attitudes and skills for self-directed, lifelong learning and scholarship. This culminates in abstract and project presentation. We report program characteristics (context and logistics) and outcomes (student engagement and experiences).

**Results:**

The Scholarly Concentrations program at Bezmiâlem began in 2014, with nearly two completed cohorts of students. In comparison to Johns Hopkins, students at Bezmiâlem begin at an earlier age (thus do not have as much prior research experience) and are subsequently evaluated for residency in terms of test scores rather than scholarship and publications, but have a similar level of intellectual curiosity and desire to take ownership of their project. Eighty-two percent of Bezmiâlem students stated the project they pursued was either their own idea or was an idea they formed after meeting with their mentor. Students at Bezmialem were more likely to choose Clinical Science projects (*p* = 0.009). Only 5% of Bezmiâlem students in end-of-course survey felt dissatisfied with the level of ownership they experienced with their project, a frequency similar to that seen by Johns Hopkins students (2%).

**Conclusions:**

Scholarly Concentrations programs play an important role in U.S. medical schools, and these programs can be successfully implemented internationally. The Scholarly Concentrations program at Johns Hopkins has been transformed to a program at Bezmiâlem in Istanbul, the first program outside North America or the European Union. When designing these programs, one must consider the context, logistics, student engagement, and outcomes. While long-term outcomes are needed, this can serve as a model for implementation elsewhere.

**Electronic supplementary material:**

The online version of this article (10.1186/s12909-019-1545-7) contains supplementary material, which is available to authorized users.

## Background

Training as a physician requires attention to not only knowledge and patient care, but also to lifelong learning and scholarship. The Liaison Committee on Medical Education (LCME) in the United States (U.S.) lists specific criteria for medical schools to meet this goal including: 1) “ensur[ing] that the medical curriculum includes self-directed learning experiences and time for independent study to allow medical students to develop the skills of lifelong learning;” and 2) “foster[ing] the intellectual challenge and spirit of inquiry appropriate to a community of scholars and provid[ing] sufficient opportunities, encouragement, and support for medical student participation in the research and other scholarly activities of its faculty” [1]. Scholarship is an important factor when considering the skills of a physician, [2] and medical schools across the U.S. have therefore incorporated independent scholarship and Scholarly Concentrations programs to help achieve these LCME objectives.

The rise of these Scholarly Concentrations programs has seen variable goals, structure, and outcomes. A 2010 review evaluated these aspects published in 39 manuscripts for programs in the United States, Canada, and the United Kingdom, and found inconsistent definition and reporting of both student and program outcomes [3]. Authors of this review describe focusing future Scholarly Concentrations program efforts on 1) creating program goals, 2) developing logic models for evaluation, 3) selecting appropriate designs of evaluation, 4) collecting multiple sources of data, 5) assessing students’ abilities, 6) obtaining IRB approval, and 7) identifying explanatory theories. Another manuscript from 2009 lists several barriers for successful implementation of these programs, including lack of preparation, faculty interest, and student time [4].

With the recognition of scholarship and lifelong learning as key factors in the growth of a physician in any country, we hypothesized we could successfully implement and transform the Johns Hopkins Scholarly Concentrations program internationally to a Turkish medical school. In particular, we discuss the Bezmiâlem Vakif University School of Medicine (Istanbul, Turkey) experiences with implementation of a Scholarly Concentrations program for medical students, including lessons learned in the challenges encountered and future directions. We rely on a theoretical framework which includes both the above descriptive analysis, and a mixed methods analysis comparing quantitative results of the Johns Hopkins and Bezmiâlem experience and themes identified by students’ comments in a convergent parallel design to understand the program’s implementation. Our experience serves as a potential model for transformation to other medical curricula outside the United States.

## Methods

### Johns Hopkins University School of Medicine

The Johns Hopkins Hospital opened in Baltimore, Maryland in 1889, and the medical school subsequently opened in 1893. The initial purposes of these institutions were to emphasize the scientific method, the incorporation of bedside teaching and laboratory research as part of the instruction, and integration of the School of Medicine with the Hospital through joint appointments. The mission of Johns Hopkins Medicine remains “to improve the health of the community and the world by setting the standard of excellence in medical education, research and clinical care. Diverse and inclusive, Johns Hopkins Medicine educates medical students, scientists, health care professionals and the public; conducts biomedical research; and provides patient-centered medicine to prevent, diagnose and treat human illness” [5].

The Johns Hopkins medical school curriculum begins after undergraduate education, and includes a traditional 4 year curriculum leading to an M.D. degree, noted by 2 years of preclinical teaching and 2 years of clinical teaching.

### Bezmiâlem Vakıf University School of Medicine

Like Johns Hopkins Medicine, Bezmiâlem Vakıf University has its origins in a hospital, and a commitment to provide care for the underserved. The hospital was established in 1845 in Istanbul, Turkey to medical services for those in need. Bezmiâlem Vakif University was then founded in 2010, and includes a School of Medicine, Dentistry, Pharmacy, and Health Sciences including Departments of Physiotherapy & Rehabilitation, Nursing, Audiology, Health Management and Nutrition & Dietetics. The mission of the university is to:

*“train healthcare professionals and scientists through innovative education models by using modern science and technology in light of the values of our civilization; to conduct research that produce real results as products and services; to provide high quality and accessible healthcare services while improving the health level of our society”* [6].

Bezmiâlem is a traditional 6-year medical school curriculum immediately after high school, leading to a M.D. degree. Bezmiâlem’s core curricular program was divided with attention to vertical integration, board exams, goals and outcomes. The goal for the overall curriculum is to “educate students who respect our national values and differences, who are competitive with international scientific research in public health, and who will meet Turkey’s contemporary needs by focusing on scientific branches of health science” [7]. As a result, the program focuses on training primary care physicians who are interested in medical research, with up to date knowledge about treatment strategies, preparation for entry into graduate medical education, and an interest in lifelong learning in Turkey and the European Union.

### Scholarly concentrations program at Johns Hopkins University

The Scholarly Concentrations program at Johns Hopkins University School of Medicine is a required component of the M.D. curriculum that provides the infrastructure and mentoring necessary for students to produce a scholarly project in an area of individual interest. The program has been present since 2009, and is similar to other Scholarly Concentrations programs across the country [4, 8–15]. By providing a faculty-mentored scholarly experience for students over their first 2 years of medical school, it encourages the acquisition of attitudes and skills for self-directed, lifelong learning and scholarship. There are 5 areas of study at Johns Hopkins University: Basic Science; Clinical Research; History of Medicine; Medical Humanities and Bioethics; and Public Health and Community Service.

In the Scholarly Concentrations Course, students are guided to perform a scholarly project and prepare a presentation of that project. In doing so, they acquire skills for self-directed learning and identify options for pursuing a scholarly career in medicine. Specific goals for the program include:

- Demonstrate the intellectual curiosity to pursue the acquisition of new knowledge and skills necessary to contribute to the scientific body of medical knowledge
- Apply scientific principles and a multidisciplinary body of scientific knowledge to create a scholarly objective or hypothesis and plan to address the objective/hypothesis.
- Present one’s own scholarship and ideas in an organized and clear manner to educate or inform colleagues and the medical community
- Demonstrate a critical self-appraisal in his or her knowledge and practice scientific inquiry, as well as receive and give constructive appraisal of scholarship to/from colleagues and other healthcare professionals
- Adhere to the highest ethical standards of judgment and conduct of scholarship

The program occupies 55.5 h in the curriculum over a period of approximately 18 months, typically in modular blocks over 3 days. There are four modules in the first year (December, February, March and May) and two modules in the second year (October and January). Students therefore must conduct almost all of the work on their scholarly project in their unscheduled time, and almost all of them do the bulk of the work in the summer between their first and second year.

The course orientation is the only time when the entire class meets as a whole. Thereafter, for each of the subsequent modules, students meet within their Concentrations with their Concentrations faculty.

So far, we have had seven cohorts of students finish the course since 2009.

### Scholarly concentrations program at Bezmiâlem Vakif University School of Medicine

**Fig. 1:**
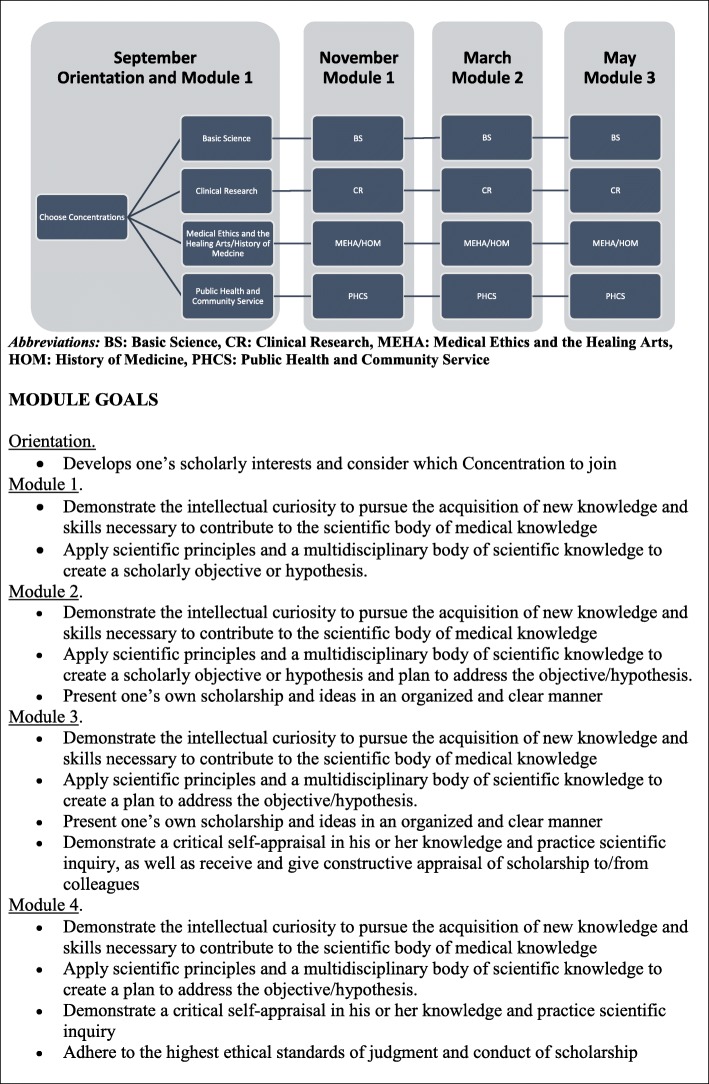
Bezmiâlem scholarly concentrations schedule, year 1

**Fig. 2:**
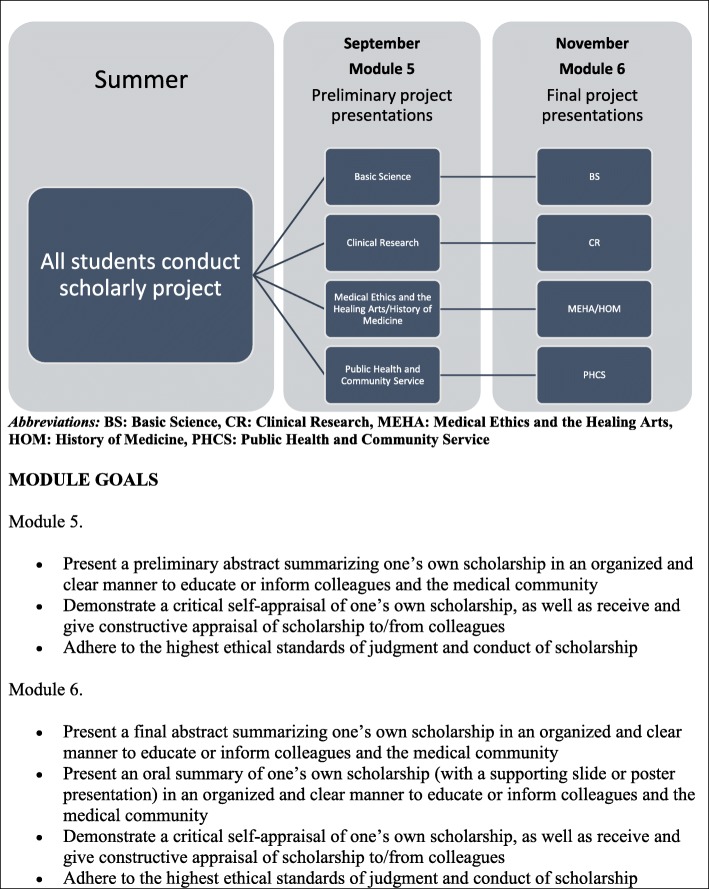
Bezmiâlem scholarly concentrations schedule, year 2

In 2014, the Johns Hopkins Scholarly Concentrations program was implemented at Bezmiâlem Vakif University School of Medicine with all students required to participate. The overall course goals and objectives are the same as the Johns Hopkins program. See Fig. 1 and Fig. 2 for a schematic display of the Bezmiâlem Scholarly Concentrations course structure and module goals, and Additional file 1 for the current schedule of didactic and small group sessions at Bezmiâlem.

#### Course orientation and module 1

In September of the first year, Bezmiâlem students have a required 1.25 h course orientation that provides an overview of the course objectives and process. Students are asked to begin to think about their scholarly interests and to consider which Concentrations they would like to join. Students are explicitly told that the Concentrations are intended to help group students into reasonably similar categories, but that they should not consider the Concentrations constraining in the choice of what scholarly project to pursue. In choosing a project, students are encouraged to think broadly about what they feel passionate about, what interests them, how they want to spend their summer and what field of medicine they wish to enter. Students are also advised about early stages of research projects, including selecting a mentor, developing a question, and searching the literature. Previous students are brought in to share their experiences.

#### Concentrations selection

Between September and November, the faculty hold office hours for students to come and discuss their interests or learn more about the Concentrations and the overlap between Concentrations. Students are asked to select a Concentration and mentor before November of their first year.

#### Finding research mentors and projects

During the course, Concentrations faculty help students identify their interests and potential mentors. At Johns Hopkins, there are three primary data sources for mentor searching: Departmental websites (if student knows basic clinical area that they want to work on), Collexis searching (Elsevier Publishing Company, Amsterdam, Netherlands) on the Johns Hopkins Library’s website (finding experts at Johns Hopkins in particular fields using MeSH terms), and the Medical Student Research Opportunities website (a Johns Hopkins-password protected data repository developed by the Scholarly Concentrations course). Similar resources are being developed at Bezmiâlem. Potential mentors at both programs are notified of course goals and asked, with each student, to sign a Mentor Agreement.

#### Modules 2–4

Modules 2–4 continue in the first year of medical school. During this time, students are given guidance regarding their own independent project. These later modules focus on human subject protection and logistical issues related to the conduct of the project.

#### Summer research

Most students elect to conduct the work for their scholarly project during the summer between first and second year for 7–9 weeks.

#### Modules 5–6

Modules 5–6 occur in the second year of medical school, and most students have had an opportunity to conduct the bulk of the work for their project in the preceding summer. These modules are spent reviewing progress, brainstorming on challenges, and discussing how to present the project in an abstract, poster, or oral presentation format.

#### Assignments

Throughout the Bezmiâlem curriculum, there are three basic written assignments (project proposal, project abstract, and poster or oral presentation), each with a preliminary and final version. Faculty provide written or oral individualized feedback to students on each assignment – both preliminary (formative feedback) and final (summative feedback) versions. Faculty refer often to Glassick’s criteria for scholarship, which can be applied to many different types of projects, when evaluating student assignments.

#### Medical student research symposium

Each student presents their scholarly project at Medical Student Research Symposium. At Johns Hopkins, all students (preclinical and clinical, including both in the Scholarly Concentrations program and already completed it) are given the opportunity to present their scholarship, and are excused from their curricular activities for this afternoon event regardless of whether or not they are presenting. At Bezmiâlem, only the preclinical students in the Scholarly Concentrations program have presented thus far. Awards are given to students and involve a judging process of posters, oral presentations, and podium presentations by course faculty.

#### Faculty leadership and organization

The Bezmiâlem course has a Director and Academic Coordinator, and each Concentration has its own faculty. In addition to support provided for the Course Director and Academic Coordinator, there is academic support for Concentrations faculty leadership. Faculty meetings are held throughout the year.

#### Course evaluation

After each Module, the students are sent a questionnaire asking them how useful they found the modules, and whether they had any recommendations for improving it. In addition, data about Scholarly Concentrations are collected by the Course Director using the following questionnaires.

1. End-of-course Student Evaluation includes 46 items covering baseline student characteristics, the students’ process during the course, the students’ experience, the project outcomes, and the impact of the course on students’ future plans.
2. Student baseline and end-of-course research self-efficacy is assessed using the Research Appraisal Inventory, a 42-item validated instrument administered through Blackboard. Originally published as the Clinical Research Appraisal Inventory (CRAI) [16], Scholarly Concentrations faculty at Johns Hopkins adapted it so that each student gets one specifically tailored to their Concentrations.

### Outcomes and analysis

We used a mixed methods design to understand outcomes after the implementation of the Scholarly Concentrations program at Bezmiâlem. Our mixed methods design was a convergent parallel design with equal parts quantitative and qualitative.

For our quantitative portion, we used the aforementioned surveys with Likert-based ratings. Early outcomes of the program included the number of students choosing each Concentration and the recognition of the perception of in-class and out-of class time. These were tabulated across programs. Mid-course and end-of-course surveys assessed attitudes about the program and mentors, and likelihood of a future research career. These formed our mid-term and long-term outcomes. Statistical comparisons between program outcomes were conducted using Fisher’s exact test for proportions. A *p*-value < 0.05 was considered statistically significant. All statistical analyses were conducted using Stata SE 14 (College Station, TX).

Our qualitative portion utilized student narrative responses on mid- and end-of-course surveys. In particular, we analyzed the mid-course response to the question, “Please express your feelings about the Concentration from the beginning in one sentence.” In addition, we analyzed the end-of-course response to the question, “What was the most positive aspect of your research experience?” Using thematic content analysis of these responses, we identified key themes that were positive and negative of the experience at mid-course, and what the largest benefit was of their research experience by end-of-course. Codes and major themes were identified by two authors (SS and MCB), using an iterative response including code exploration, identification of themes, and refinement and finalization of themes. Discrepancies were resolved through discussion to reach consensus.

## Results

### Transforming context of scholarly concentrations program

The goals of transforming the Scholarly Concentrations program in 2014 to Bezmiâlem Vakif University School of Medicine were to not only to educate Turkish medical students in the information field of Medicine, but also to educate students on how to question and produce this information. Johns Hopkins University Scholarly Concentrations leaders advise on the development of the curriculum, deliver several lecture and small group sessions, meet individually and in groups with students, and collaborate with Bezmiâlem Vakif University faculty on methods to transform the curriculum in the university setting. This has included two international planning trips, and eight international teaching trips between 2015 and 2017 covering both the first iteration of the course and Bezmiâlem faculty development.

Bezmiâlem was not a simple and passive user of this Scholarly Concentrations program, but instead used it to enhance the interest in scientific research among the students. This included increasing the number of dedicated lectures in the first 2 years getting to prepare for the Scholarly Concentrations experience, and addition of animal use in experimental research courses.

Several similarities and differences have been observed between the Johns Hopkins and Bezmiâlem programs, students, and mentors. (Table 1) The Scholarly Concentrations program at each institution receives institutional support from the respective school’s Vice Dean for Undergraduate Medical Education. This brings faculty salary support and protected effort for Scholarly Concentrations faculty, as well as priority in scheduling the curriculum. The Scholarly Concentrations program at each institution depends on faculty mentorship of the students in a student-centered environment. Johns Hopkins is known for their research expertise, leading the country in National Institutes of Health research dollars, and having the faculty to support this mission. This level of faculty engagement in the research process is likely not present at most other European academic medical centers, and can present challenges when finding mentors and projects for students. However, Bezmiâlem has one of the highest averages in Turkey in number of articles published per academic staff at 1.74, and the current ratio of academic personnel per student is 1:7, above the world standard [7].

**Table 1:** Differences in environment and context for the scholarly concentrations course

	Johns Hopkins University	Bezmiâlem Vakif University
Support from Leadership	High	High
Faculty Mentors	> 3000 medical school faculty mostly involved in research	455 academic personnel involved in clinical work and research
External Student Motivation (Instrumental Value of Course)	Publications valued for residency applications	Exam scores valued for future career success
English Proficiency	High	Developed during medical school for most
Characteristics of Students	Already graduated college, many with research experience	Recently graduated high school, very few with research experience
Number of Students	~ 100 (not in PhD track)	~ 100
Internal Curiosity	High	High

Students at each institution also have similarities and differences. As a traditional US medical school program, Johns Hopkins has students who started medical school after an undergraduate degree, often with additional work, life or coursework experiences in gap years prior to starting medical school. Bezmiâlem’s students join the university after high school, without as long an experience in research or other advanced training. The average age of entering Johns Hopkins student is 23, with 47.5% women, and 5% international students. The average age of entering Bezmiâlem students is 19, with 60.7% women, and the majority are originally from Turkey; there are no students at Bezmiâlem from the U.S. The cultural differences in both society and education between the U.S. and Turkey cannot be overlooked. In terms of education, Bezmiâlem students have come from prior training where test scores are the driving force for advancement. This makes implementation of a program such as Scholarly Concentrations, which stresses process of acquiring new knowledge rather than the knowledge itself, have additional challenges. However, students at both institutions possess intellectual curiosity and a desire to understand medicine and make an impact in the field.

In addition, students take ownership of their projects, while still working in conjunction with their faculty mentor. Eighty-two percent of Bezmiâlem students stated the project they pursued was either their own idea or was an idea they formed after meeting with their mentor, while 18% of students pursued a project suggested primarily by faculty.

### Transforming logistics of scholarly concentrations program

Timing of each module depends on the underlying structure of the rest of the curriculum, as well as understanding the developmental progression of the students. At Johns Hopkins, students are required to attend Scholarly Concentrations unless they are part of the Medical Scientist Training Program (MSTP). Students in the MSTP have a separate curriculum to ease transition to their PhD years.

At Bezmiâlem, this is not only a relatively new medical school but also a new curriculum. While all students at Bezmiâlem participate in this program, having students understand the importance of Scholarly Concentrations in their overall medical school curriculum has been one challenge.

Transforming the module timing also has challenges. At Johns Hopkins, we have scheduled Scholarly Concentrations blocks for nine total hours spread out over 3 days. At Bezmiâlem, the same time is allotted, but these are concentrated in one or two days. There are also opportunities to contact the Scholarly Concentrations Advisor, and communicate by email or other methods on progress between the blocks. Both programs’ blocks occur approximately every 2 months in the first year, and every 4 months in the second year.

### Student engagement in the scholarly concentrations programs

Faculty mentorship and advising is a hallmark of Scholarly Concentrations at each program. Students present to each other and course faculty, and receive feedback from faculty and peers. In addition, student experiences from past years in choosing projects and mentors have been key parts of the success of the Johns Hopkins program.

Since Bezmiâlem is only in its second year, the Scholarly Concentrations alumni experience from Bezmiâlem students who completed the program is limited. As a result, the Johns Hopkins program has had four students present their Scholarly Concentrations research and its obstacles to the Bezmiâlem students, allowing an open student-centric dialogue of successes and potential keys to that success. This year, Bezmiâlem students who completed the program have started to share this wealth of information.

### Transforming outcomes of scholarly concentrations program

While the exact curriculum and its timing have differences in the Johns Hopkins and Bezmiâlem programs, the expected outcomes and experienced outcomes are similar. Both programs stress passion for discovery, and student engagement in the process and their project. Students are expected to take ownership in a project, and faculty mentors and advisors guide them to project completion.

Students at both programs are expected to complete their project and present in abstract form. In addition, students are expected to present in poster, oral, or podium presentation at Medical Student Research Symposium. At Bezmiâlem, the first Medical Student Research Symposium occurred March 2017, and included presentations, awards for outstanding projects, and a vibrant atmosphere of faculty-student interactions.

### Outcomes observed

As a result of students’ engagement in their own education and projects, students at each university choose their own areas of interest, and not surprisingly, Concentrations are therefore not equally subscribed at either Johns Hopkins or Bezmiâlem (Table 2). The study areas in Bezmiâlem only included Basic Science, Clinical Research, History of Medicine/Medical Ethics and the Healing Arts, and Public Health. The distribution of student choices of Concentration was different across programs (*p* = 0.009). At Johns Hopkins, Clinical Research (47%) and Public Health/Community Service (28%) have been consistently the most popular choices while at Bezmiâlem, Basic Science (20%) and Clinical Research (53%) have been the most common.

**Table 2:** Number of students enrolled in each concentration

	Johns Hopkins Cohort							TOTAL Johns Hopkins	TOTAL Bezmiâlem	*p*-value
Concentration	2009	2010	2011	2012	2013	2014	2015	**%**	**%**	
Basic Science	8	24	9	6	14	11	12	84 (11%)	21 (20%)	0.009
Clinical Research	57	53	48	54	43	50	55	360 (47%)	56 (53%)	
History of Medicine	8	4	10	5	10	11	4	52 (7%)	9 (8%)	
Medical Ethics and the Healing Arts	5	10	9	8	8	7	11	58 (8%)		
Public Health and Community Service	40	30	31	27	29	31	25	213 (28%)	20 (19%)	

*p-value by Fisher’s exact test comparing proportion of students in each Concentration across programs (History of Medicine and Medical Ethics and the Healing Arts combined for the Johns Hopkins cohort

Perception of the time in class differed between the programs. In end-of-course survey, 39% of students at Bezmiâlem felt they could needed more time for in-class work, whereas 35% felt they needed less in-class time; only 26% felt it was the right amount of time. This is significantly different (*p* < 0.001) from the Johns Hopkins experience, where 50% of students felt in-class time was the correct amount, and 48% felt needed less in-class time. Only 2% of Johns Hopkins students felt they needed more in-class time. Similar distributions were also seen for out-of-class time, with 26% of Bezmiâlem students feeling they had the right amount of out-of-class time, and 74% of Johns Hopkins students feeling the same way (*p* < 0.001).

The first cohort of Bezmiâlem students identified several themes about their involvement in the program. (Table 3) Positive themes include development of new skills and appreciation of science and discovery. Negative themes include time commitment, mentor and project obstacles. In addition, students at Bezmiâlem identified the following as the top themes of their research projects: a sense of self-efficacy, a sense of being involved in professional or important work, presentation skill development, and research skill development (Table 4). Since it serves the main aim of Bezmiâlem Vakif University, the program has raised the enthusiasm among both faculty and medical students. The highest rated item by students is the ability to work with these faculty mentors. In addition, students appreciate the ability to work on a project they helped develop. Only 5% of Bezmiâlem students in end-of-course survey felt dissatisfied with the level of ownership they experienced with their project, a frequency similar to that seen by Johns Hopkins students (2%). 89% of Bezmiâlem students stated their project helped them experience the excitement of discovering something new.

**Table 3:** Scholarly concentrations themes experienced at Bezmiâlem at mid-course

	Representative Quotes from Students^a^
Positive Themes	
New Skills Obtained in Literature Review, Ethics	“I learned how to review the literature.”
	“I learned how to prepare the application for the ethical committee.”
Appreciation of Science and Discovery	“I learned how to work in the laboratory.”
	“I learned the importance of scientific research projects.”
Negative Themes	
Personal Time Commitment Required	“I didn’t know that it would need that much effort.”
	“The given time frame isn’t suitable for everyone.”
Mentor Concerns	“My mentor left and I have a new mentor.”
Project Obstacles	“There are insufficient number of patients at the particular time.”
Cultural Issues Around Research in Turkey	“I have difficulties in understanding English literature.”

^a^In response to prompt, “Please express your feelings about the Concentration from the beginning in one sentence”

**Table 4:** Scholarly concentrations themes experienced at Bezmiâlem at end-of-course

	Representative Quotes from Students^a^
Sense of Self-Efficacy	“Learning that we can do research ourselves.”
	“When I started this project I did not believe that I would be able to complete the project. But by the end I understood that I can make it happen.”
	“To begin to believe in myself and to gain confidence when it comes to scientific project.”
Sense of Being Involved in Professional or Important Work	“The chance to contribute knowledge to the world was one of the most satisfying parts for me.”
	“The most valuable part was having a hands on experience about a professional project.”
	“Being part of science during medical school.”
Presentation Skill Development	“Learning how to present a project.”
Research Skill Development	“Understanding the steps of making research.”
	“Writing article for scientific project.”
	“My mentor left and I have a new mentor.”
	“To be able to evaluate statistical information.”
	“Having a knowledge about the procedure of how to make a clinical research.”

^a^In response to prompt, “What was the most positive aspect of your research experience?”

In final evaluation, 82% of Bezmiâlem students said they were likely to pursue scholarly work in the future, due to the impact of their Scholarly Concentrations project. This aligns closely with the Johns Hopkins experience, where 89% of Johns Hopkins students expressed likelihood of future scholarship.

### Obstacles faced in this process

The sections above discuss how the Johns Hopkins program was transformed to a Bezmiâlem program. With implementation of a new program, obstacles always develop. These required flexibility by both Johns Hopkins and Bezmiâlem faculty, and strategies to overcome these obstacles. The main obstacles we encountered were 1) Faculty development in terms of training mentors; 2) Student engagement in terms of changing expectations for medical education including how this impacts their residency application; and 3) Appreciation of the impact on this curriculum on the European Credit Transfer and Accumulation System.

In addition to having Johns Hopkins faculty continue to work with Bezmiâlem both in person and via electronic means, we have developed several other methods to overcome these challenges. For mentorship, we developed specific sessions to review expectations and opportunities for local mentors, invited mentors and potential mentors to the Medical Student Research Day, and rewarded highest performing mentors with awards at the annual Doctor’s Day reception (i.e. top-down approach). We also reviewed the roles of a mentee with the students, and included both positive and negative mentee models plus strategies for navigating a relationship (i.e. bottom-up approach). To generate engagement in a new curriculum, we took a student-centric approach, where prior students discussed the benefits and success of the program as they approached future work and studies. Students now accepted at Bezmiâlem have this program as an expectation, and many choose to matriculate at Bezmiâlem explicitly for this program. Finally, for the credit system, having buy-in from University leadership helped navigate the complexity that can develop from having such a program, that otherwise does not fit in the traditional model. Undoubtedly, other obstacles will develop and having faculty that are invested in the program and knowledgeable about the process is essential in overcoming these future obstacles.

## Discussion

The Scholarly Concentrations program at Johns Hopkins University School of Medicine has been a successful way to introduce students to the process of discovery, and develop their passion for scholarship and work with a mentor. This program has now been transformed to Bezmiâlem Vakif University School of Medicine in Istanbul, with a second iteration of the program starting now. To our knowledge, this is the first time this has been transformed to a program outside of North America or the European Union, and can serve as a model for successful implementation elsewhere.

We found there were barriers to transforming the context, logistics, and outcomes of a U.S. medical education program to a setting outside the U.S. However, the interest in science, the passion for discovery, and ownership of projects by the students were present at both institutions. This is not possible without institutional support, and dedication to the process by faculty, students, and project mentors, and also active engagement by the Bezmiâlem faculty to make the program fit their own particular needs. We expect logistics of the course and long-term outcomes to continue to be refined and improved upon as we understand the exact needs of the students in this experience.

As for which future planning and outcomes to focus on, the Bezmiâlem program will follow lessons learned from the Johns Hopkins program and other Scholarly Concentrations programs elsewhere. Burk-Rafel performed a qualitative analysis of the top 25 ranked U.S. medical schools’ Scholarly Concentration programs and found that a data-driven approach can influence Concentration selection by students [17]. For the Bezmiâlem program, this has been performed informally, as reflected by a combined History of Medicine/Medical Ethics and the Healing Arts Concentration, though more formal student feedback on Concentration and topic selection will guide this further in future years. In the Johns Hopkins program, we have explored models of identifying successful mentoring relationships, [18] and similar models will be conducted in the examined the Bezmiâlem experience. Finally, our long-term outcomes should be cognizant of the rigor needed to assess the impact of Scholarly Concentrations programs, [3] including such items as research productivity and the predictors of that research productivity [19]. Long-term outcomes at Bezmiâlem, including careers in research and productivity, will be investigated through additional surveys and focus groups.

While the concept of transformation of Scholarly Concentrations programs to Turkey is relatively novel, other programs have been seen worldwide to teach medical students about research. Reinders reports an evaluation of medical student productivity in the Netherlands. While this was not a formal program of research, they find that students who participated more in research activities and articles during medical school were more likely to publish after graduation [20]. Elwood described the honors year in community health in England, and found students had high satisfaction for the program and increased their research skills [21]. Remes describes the research experiences of students in Finland. This also was not a formal research program; however, they find that students pursued research out of scientific curiosity and a desire for residency. They conclude, “Medical students should be encouraged to carry out research work, because they thus learn critical and logical thinking” [22]. Finally, medical student research programs in Canada have been successful for years [23, 24]. Even though there are successful models elsewhere worldwide, ours is the first program to use a US-based format elsewhere.

Limitations of this study do exist. Ideally, we would wait for additional years of data that could be connected to specifics related to the implementation in this new curriculum, as we are not able to yet report long-term outcomes beyond Concentration selection and future potential interest in research. However, non-US programs may still be interested in the lessons we have learned so far before these long-term outcomes. Next steps may assess the process itself, faculty evaluation of individual students and their needs, or the passion for discovery. In addition, we only report transformation at one institution. However, the steps learned here may be used in other settings, and set a framework for points to consider.

## Conclusions

In conclusion, a Scholarly Concentrations program can meet the needs of both US students and students internationally. Further long-term outcomes and other programs’ implementation are needed.

## Additional file

Additional file 1: Bezmiâlem Vakif University Scholarly Concentrations Schedule. This is the current schedule of didactic and small group sessions for Bezmiâlem Scholarly Concentrations. (DOCX 26 kb)

## Abbreviations

CRAI: Clinical Research Appraisal Inventory
LCME: Liaison Committee on Medical Education
MSTP: Medical Scientist Training Program
SC: Scholarly Concentrations
