# Supplementary material for: Transforming a U.S. scholarly concentrations program internationally: lessons learned
Source: BMC Med Educ. 2019 Apr 25;19:115. doi: 10.1186/s12909-019-1545-7 (PMC6485131; doi:10.1186/s12909-019-1545-7)
Supplement: Supplementary file 1 — Bezmiâlem Vakif University Scholarly Concentrations Schedule. This is the current schedule of didactic and small group sessions for Bezmiâlem Scholarly Concentrations. (DOCX 26 kb) [file 12909_2019_1545_MOESM1_ESM.docx]

**Transforming a U.S. Scholarly Concentrations Program Internationally: Lessons Learned**

**Supplemental Material**

Supplement 1: Bezmiâlem Vakif University Scholarly Concentrations Schedule

**Supplement 1: Bezmiâlem Vakif University Scholarly Concentrations Schedule**

**MODULE 1**

**September of 1st year of Program--Day 1**

09:00-09:30 **Lecture: Opening**

09:30-10:00 **Lecture: Scholarly Concentrations, Step by Step**

10:00-10:15 **Lecture: Overview of Scholarly Concentrations**

10:15-11:00 **Lecture: Why Scientific Projects are Necessary?**

*11:00-11:15 Break*

11:15-12:15 **Lecture: Stages of a Research Project**

*12:30-13:30 Lunch*

13:30-14:00 **Lecture: Finding the Research Question**

14:00-14:30 **Lecture: How Can You Work With Your Mentor?**

14:30-15:00 **Lecture: Things to Know Just Before the Start**

*15:00-15:15 Break*

15:15-16:00 **Large Group Discussion: Questions & Answers**

**September of 1st year of Program--Day 2**

09:00-09:45 **Lecture: Evidence Based Medicine**

09:45-10:15 **Lecture: Literature Search Techniques**

*10:15-10:30 Break*

10:30-12:00 **Panel Discussion: Previous Student Experiences on Projects**

*12:00-14:00 Lunch*

**MODULE 2**

**November of 1st Year of Program**

09:00-09:20 **Lecture: Introduction**

09:20-09:35 **Lecture: From Module 2 to Module 3**

*09:35-10:00 Break*

10:00-10:30 **Lecture: Sample Size Calculation**

10:30-10:50 **Lecture: IRB Applications**

10:50-11:10 **Lecture: How to Work With Mentors in Designing the Study**

*11:10-11:20 Break*

11:20-11:40 **Lecture: The Most Common Types of Research Problems that Students Do**

11:40-12:00 **Lecture: Next Steps**

*12:00-13:00 Lunch*

13:00-15:00 **Small Groups: Presentations by Students on Preliminary Project Ideas (2 slides)**

**MODULE 3**

**March of 1st Year of Program**

09:00-09:20 **Lecture: Introduction**

09:20-09:40 **Lecture: From Module 3 to Module 4**

09:40-10:10 **Lecture: Qualitative Methods--Data Collection and Analysis**

10:10-10:40 **Lecture: Qualitative and Quantitative Methods--Survey Design**

*10:40-11:00 Break*

11:00-11:30 **Lecture: Quantitative Methods--Choosing Variables in Research**

11:30-12:00 **Lecture: Quantitative Methods--Practical Guide to Choosing a Statistical Test**

*12:00-13:00 Lunch*

13:00-13:20 **Lecture: Application for Budget**

13:20-13:40 **Large Group Discussion: IRB Applications**

13:40-15:20 **Small Groups:** **Student Presentations of Preliminary Project Proposals**

*15:20-15:40 Break*

15:40-17:10 **Small Groups:** **Student Presentations of Preliminary Project Proposals**

**MODULE 4**

**May of 1^st^ Year of Program-- Day 1**

09:00-09:15 **Lecture: Introduction**

09:15-09:30 **Lecture: From Module 4 to Summer to Module 5**

09:30-10:15 **Lecture: Overcoming Obstacles in a Scholarly Concentrations Project**

*10:15-10:30 Break*

10:30-11:00 **Lecture: Following the Protocol**

11:00-11:20 **Large Group** **Discussion: Budget Application Process**

11:20-12:15  **Previous Student Experiences on IRB, Budget, Data Collection**

*12:15-13:30 Lunch*

13:30-17:00 **Small Groups:** **Student Presentations of Final Project Proposals**

**May of 1^st^ Year of Program-- Day 2**

09:00-12:00 **Small Groups: Workshop to Identify Potential Obstacles and Solutions**

**MODULE 5**

**September of 2^nd^ Year of Program-- Day 1**

09:00-09:30 **Lecture: Introduction**

09:30-09:45 **Lecture: From Module 5 to Module 6**

09:45-10:15 **Lecture: From Data to the Results, Materials and Methods**

*10:15-10:30 Break*

10:30-11:00 **Lecture: Redaction of the Results**

11:00-11:30 **Lecture: Considerations During Presentations**

11:30-12:00 **Large Group Discussion**

*12:00-13:00 Lunch*

13:00-14:30 **Small Group Discussion: Evaluation of Scientific Project Examples**

*14:30-15:45 Break*

15:45-17:00 **Small Group Discussion: Evaluation of Scientific Project Examples**

**September of 2^nd^ Year of Program-- Day 2**

09:00-09:30 **Lecture: Preparing the Abstract**

09:30-10:30 **Workshop: Preparing the Abstract: Discussion and Exercise Part 1 (Title and Introduction)**

*10:30-11:00 Break*

11:00-12:00 **Workshop: Preparing the Abstract: Discussion and Exercise Part 2 (Methods)**

*12:00-13:00 Lunch*

13:00-14:00 **Panel Discussion: Previous Student Experiences on Abstracts and Presentations**

**MODULE 6**

**November of 2^nd^ Year of Program—Day 1**

09:00-16:00 **SC Faculty Meet Individually with Students**

**November of 2^nd^ Year of Program—Day 2**

08:45-09:00 **Lecture: Introduction**

09:00-10:30**Workshop: Preparing the Abstract: Discussion and Exercise Part 3 (Results)**

*10:30-11:00 Break*

11:00-12:30 **Workshop: Preparing the Abstract: Discussion and Exercise Part 4 (Discussion)**

*12:30-13:30* *Lunch*

13:30-14:00 **Lecture:** **How to Write an Article**

14:00-14:30 **Lecture:** **How to Prepare a Poster Presentation**

14:30-15:00  **Lecture:** **How to Give an Oral Presentation**

**Medical Student Research Symposium**

**March of 2^nd^ Year of Program**

09:00-09:20  **Lecture: Introduction**

09:20-10:20**Podium Presentations + Discussion**

*10:20-10:40 Break*

10:40-11:40 **Poster Presentations + Discussion**

*11:40-13:00* *Lunch*

13:00-14:20 **Breakout Rooms: Oral Presentations**

14:30-16:00 **Award Ceremony/Medical Festival**
